# Characterization and use of HapT1-derived homologous tumors as a preclinical model to evaluate therapeutic efficacy of drugs against pancreatic tumor desmoplasia

**DOI:** 10.18632/oncotarget.9729

**Published:** 2016-05-31

**Authors:** Sujit Suklabaidya, Biswajit Das, Syed Azmal Ali, Sumeet Jain, Sharada Swaminathan, Ashok K. Mohanty, Susen K. Panda, Pujarini Dash, Subhankar Chakraborty, Surinder K. Batra, Shantibhusan Senapati

**Affiliations:** ^1^ Tumor Microenvironment and Animal Models Laboratory, Department of Translational Research, Institute of Life Sciences, Bhubaneswar, Odisha, India; ^2^ Manipal University, Manipal, Karnataka, India; ^3^ Proteomics and Structural Biology Laboratory, Animal Biotechnology Department, National Diary Research Institute, Haryana, India; ^4^ Department of Bioengineering, School of Chemical and Biotechnology, SASTRA University, Thanjavur, Tamil Nadu, India; ^5^ Department of Veterinary Pathology, Odisha University of Agriculture and Technology, Bhubaneswar, Odisha, India; ^6^ Mayo Clinic, Division of Gastroenterology and Hepatology, Rochester, Minnesota, MN, USA; ^7^ Department of Biochemistry and Molecular Biology, Buffett Cancer Center, Eppley Institute for Cancer Research, University of Nebraska Medical Center, Omaha, NE, USA

**Keywords:** pancreatic cancer, desmoplasia, HapT1, pancreatic stellate cells, hamster homologous orthotopic model

## Abstract

Desmoplasia in human pancreatic cancer (PC) promotes cancer progression and hinders effective drug delivery. The objectives of this study were to characterize a homologous orthotopic model of PC in Syrian golden hamster and investigate the effect of anti-fibrotic (pirfenidone), antioxidant (N-acetyl cysteine, NAC) and anti-addiction (disulfiram, DSF) drugs on desmoplasia and tumor growth in this model. The HapT1 PC cells when implanted orthotopically into hamsters formed tumors with morphological, cellular and molecular similarities to human PC. Protein profiling of activated hamster pancreatic stellate cells (ha-PSCs) revealed expression of proteins involved in fibrosis, cancer cells growth and metastasis. Pirfenidone, suppressed growth of HapT1 cells and the desmoplastic response *in vivo*; these effects were enhanced by co-administration of NAC. Disulfiram alone or in combination with copper (Cu) was toxic to HapT1 cells and PSCs *in vitro*; but co-administration of DSF and Cu accelerated growth of HapT1 cells *in vivo*. Moreover, DSF had no effect on tumor-associated desmoplasia. Overall, this study identifies HapT1-derived orthotopic tumors as a useful model to study desmoplasia and tumor-directed therapeutics in PC. Pirfenidone in combination with NAC could be a novel combination therapy for PC and warrants investigation in human subjects.

## INTRODUCTION

Pancreatic ductal adenocarcinoma (PDAC) is the fourth common cause of cancer related deaths [1]. It has a 5 year survival rate of 6% and it is predicted to be the second leading cause of cancer deaths by 2030 [2]. PDAC stands out from other epithelial neoplasms as it exhibits desmoplasia which manifests as overproduction of extracellular matrix and extensive proliferation of myofibroblast like cells. Desmoplasia arises from the activation of pancreatic stellate cells (PSCs) that constitute 4–7% of the total cell population of normal pancreas [3, 4]. Although PSCs remain quiescent normally, it has been shown that pancreatic cancer cells (PCCs) activate PSCs in a paracrine fashion and as a result PSCs differentiate into α-SMA positive myofibroblast-like cells that produce high amounts of extracellular matrix (ECM) proteins, particularly collgen-I and fibronectin [5]. The crosstalk between PCCs and PSCs has been associated with tumor progression and metastasis [6]. Desmoplasia in PDAC contributes to chemoresistance which in turn results in poor prognosis [3]. Studying the role of PSCs in PDAC is indispensable and so it is necessary to develop suitable *in vitro* and *in vivo* models that closely resemble the tumor associated desmoplasia seen in humans.

While previous studies have employed PSCs co-cultured with PC cells or *in vivo* models of genetically modified mice or xenografts, these models have several limitations that make them unsuitable to study novel therapeutics against PC. For example, xenograft models lack all the cellular components of a fibrotic tumor because of the absence of an effective immune system. Genetically modified mouse models have long periods of tumor latency that limits their usefulness in testing new therapeutics. Therefore, there is a need for new animal models that overcome the shortcomings of existing models, particularly to test new treatment strategies.

The chemically induced model for PDAC in Syrian golden hamsters (*Mesocricetus auratus*) was shown to be similar in morphologic appearance and biological behaviour to that seen in humans [7]. Moreover, the chemically induced hamster models using N-nitroso-bis(2-oxopropyl)amine (BOP) have also shown to harbour point mutations in the *K-ras* gene [8]. Different labs have established and characterized multiple PC cell lines from carcinogen induced pancreatic tumor in hamsters [9, 10]. Some of these cell lines have been frequently used to generate allograft tumors in Syrian hamsters. Particularly, the commercially available HapT1 hamster PC cell line derived from DIPN-induced pancreatic tumor has been widely used as an *in vitro* model in PC related studies [10]. Carcinogen induced hamster PC shows desmoplastic reactions; however, till date no studies have reported the characterization and/or use of hamster PC model as a pre-clinical model for cancer-associated desmoplasia.

As discussed above, cross talk between PSCs and PCCs provides significant contribution to the malignant behaviours of cancer cells and resistance to established therapy for PC. Therefore, it is a common belief that pharmaceutically reducing the activated PSCs number in the tumor tissue either by killing (stromal ablation) or by inactivating (stromal normalisation) would enhance the clinical efficacy of conventional chemotherapy. Pirfenidone (5-methyl-1-phenyl-2-[1H]-pyridone), a drug approved for idiopathic pulmonary fibrosis (IPF) has shown encouraging anti-fibrotic effects in a mouse model of PC [11]. Pirfenidone decreases PSCs proliferation, invasion and migration *in vitro* and inhibits subcutaneous tumor formation in mice co-transplanted with PCCs and PSCs [11]. A recent study has shown a better patient outcome with combined therapy of pirfenidone and N-acetylcystein (NAC) in advanced idiopathic pulmonary fibrosis patients [12]. Moreover, NAC sensitizes human PCCs to gemcitabine by inhibiting NFκB pathway [13]; and cultivation of PSCs on extracellular matrix proteins and treatment with NAC induced deactivation of PSCs and reduced their proliferation and fibrogenic property [14]. However, till date no study has been reported that evaluate the effect of pirfenidone and NAC co-administration on desmoplastic PC. At the same time, like pirfenidone there are multiple other drugs which are approved for certain diseases other than cancer, but could have significant use in cancer therapy. Disulfiram (1-[diethylthiocarbamoyldisulfanyl]-N,N-diethyl-methanethioamide) a FDA approved drug has been reported to have cytotoxic effect against variety of cancer cells including PC, while sparing normal cells [15, 16]. In preclinical studies, Disulfiram (DSF) in combination with copper ions has been shown to inhibit or suppress NFκB signalling, proteasome activity, aldehyde dehydrogenase activity, and antioxidant levels in cancer cells [15–18]. To date, however, no studies have analysed the effects of DSF on PSCs. Therefore, the major goals of this study were to establish and characterise a hamster PC model that shows the desmoplastic reactions similar to human PDAC, and to evaluate whether pirfenidone (in presence or absence of NAC) and DSF (in presence or absence of Cu) could reduce activated PSCs growth and supress desmoplasia *in vitro* and *in vivo*.

## RESULTS

### Morphology and composition of HapT1 primary and metastatic tumors

**Figure 1:**
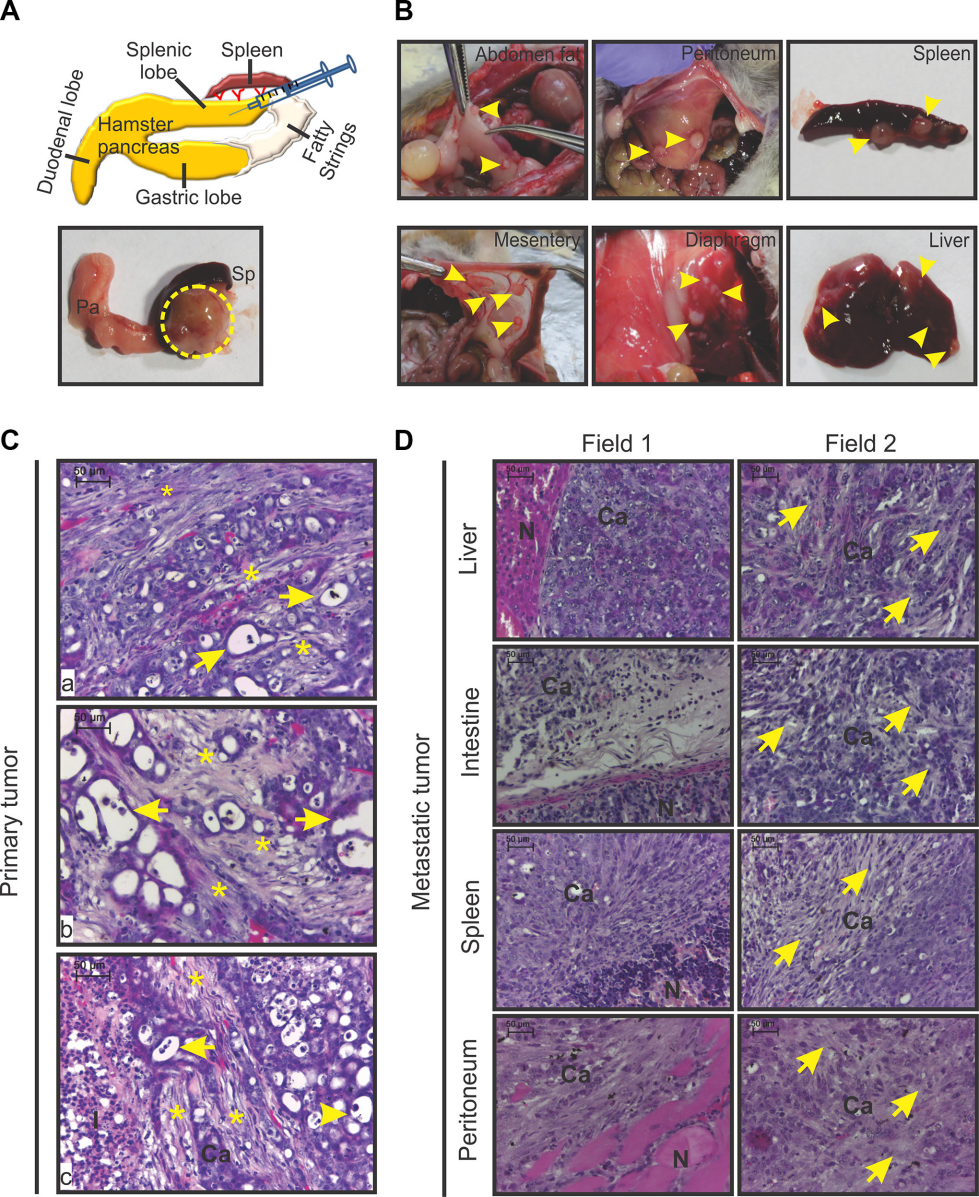
Gross morphology and histopathology of HapT1 orthotopic tumor (**A**) Diagram of the orthotopic implantation of HapT1 cancer cells in hamster pancreases. Representative macroscopic view of a HapT1 orthotopic tumor present inside the splenic lobe of a hamster pancreas (dotted circle). The splenic lobe of the pancreas (pa) and spleen (sp) are also shown in the image. (**B**) Representative gross images of various organs that showed metastatic tumor growth are shown (arrowhead). (**C**) Representative images of H&E stained sections of HapT1 orthotopic primary tumors from three different tumor sections (a, b, and c). An image from primary pancreatic tumor sections shows the presence of pseudo-duct-like structures (arrow) and deposition of dense extracellular matrix proteins/desmoplstic reaction around them (star). At some places, extracellular matrix proteins act as a barrier between tumor-infiltrated immune cells and cancer cells (c). (**D**) H&E stained sections of HapT1 metastatic tumors clearly show the presence of a desmoplastic reaction within the tumor stroma of metastatic tumors (arrowhead). (N) normal tissue area; (Ca) cancerous regions; (I) immune cells (scale bar= 50 μm).

Necropsy of all the animals in this study showed 100% tumor uptake. All the tumors were firm, round, and slightly reddish in colour (Figure 1A). Further, all animals had incurred metastasis to one or multiple sites/organs (Figure 1B). Microscopically, the primary tumors were found to consist of multiple duct-like structures (pseudo ducts; Figure 1C), and the proportion of duct-like structures was greater in 24-day-old tumors compared with the 14-day-old tumors (data not shown). The tumors had centrally located necrotic zones and peripheral proliferative zones. Necrotic regions were laden with infiltrated immune cells (data not shown). Some immune cells were also visible in the non-necrotic regions of all the tumors. Spindle-shaped myofibroblast-like cells and the deposited extracellular matrix were clearly visible around all the individual duct-like structures (Figure 1C). At multiple places, the stromal structures surrounding the cancer cells acted as a barrier between infiltrated immune cells and cancer cells (Figure 1C). As reported for human PC, the secondary or metastatic tumors harvested from multiple hamsters clearly showed the presence of fibrotic stroma (Figure 1D; [19]). Irrespective of the site of metastasis, all the metastatic tumors were desmoplastic; activated myofibroblast-like cells and immune cells were also present.

**Figure 2:**
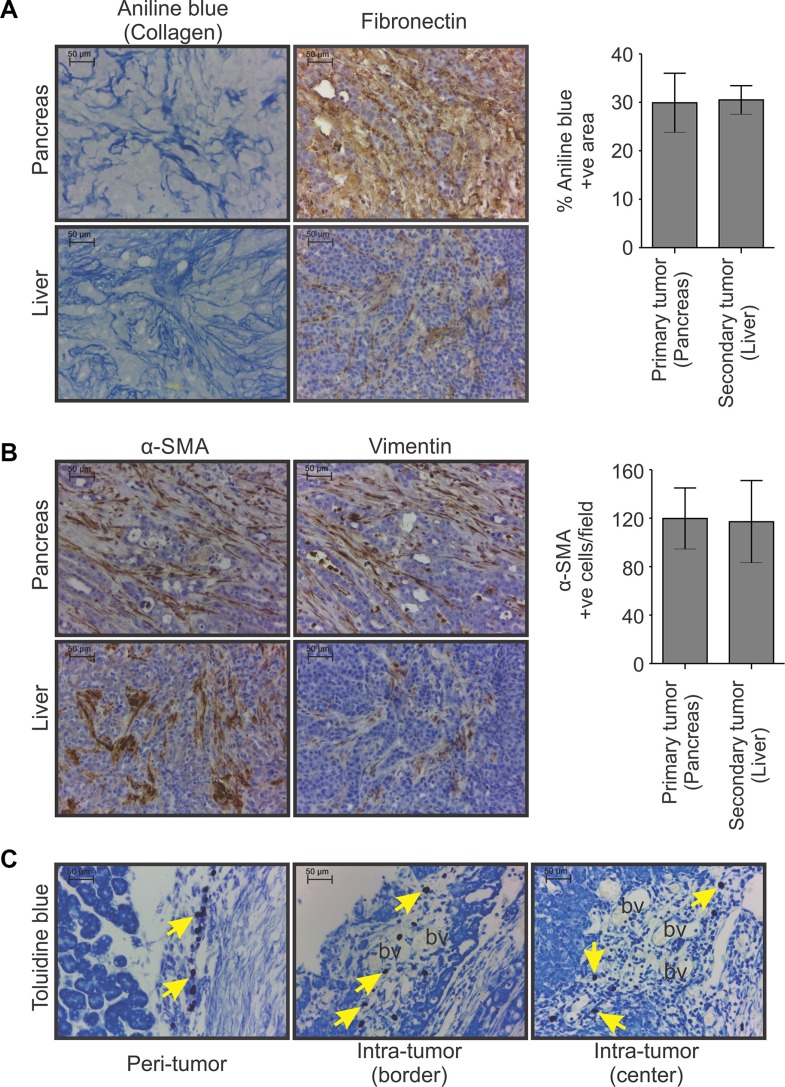
Non-cellular and cellular components of HapT1 orthotopic tumor (**A**) Representative images of primary and metastatic tumor tissue sections (liver) showing the presence of collagen and fibronectin, as detected by aniline blue staining and IHC analysis, respectively (scale bar = 50 μm). Morphometric analysis of images obtained from aniline blue stained sections shows the extent of collagen deposition in primary (pancreas) and corresponding metastatic tumors (liver). The percentage of aniline blue stained area estimation was done in tissues obtained from two different animals (with liver metastasis). Presented data obtained from one animal (*n* = 5; five different fields/tissue) shows the representative pattern of collagen deposition in corresponding primary and metastatic tumors. Data presented as mean ± SE. (**B**) IHC analysis for α-SMA and vimentin as markers of activated myofibroblast-like cells followed by quantification of α-SMA positive cells shows the presence of a similar number of activated myofibroblast-like cells in both primary and corresponding metastatic tumors. Presented data obtained from one animal (*n* = 5; five different fields/tissue) show the representative pattern of the abundance of α-SMA positive cells in corresponding primary and metastatic tumors (scale bar = 50 μm). Data presented as mean ± SE. (**C**) Representative images of toluidine blue-stained primary tumor sections showing the presence of mast cells (violet color; arrows) in peri-tumor, intra-tumor border and intra-tumor center regions of HapT1 orthotopic tumors (scale bar = 50 μm). Most of the mast cells were present closer to the blood vessels (bv).

For further molecular and cellular characterization of the desmoplastic reactions present in HapT1 tumors, this study examined the expression of two major extracellular matrix proteins (*i.e.*, collagen and fibronectin) and two important cell types known to promote PC-associated fibrosis (*i.e.*, myofibroblast-like and mast cells). Staining with aniline blue (one component of MS trichome stain) showed dense collagen deposition in all the primary and metastatic tumors (Figure 2A; data not shown). However, the normal pancreatic tissue present in closer proximity to the primary tumors had markedly low levels of collagen (Supplementary Figure S1). Figure 2A shows immunohistochemical (IHC) staining of tumors derived from HapT1 cell lines and confirms the area that stained positive for collagen also stained positive for fibronectin. Moreover, fibronectin was also present in the metastatic tumors (Figure 2A; data not shown). IHC analysis of tumor sections also revealed that HapT1tumors contained myofibroblast-like cells, which is based on the fact that they stained positive for α-SMA and vimentin. The myofibroblast-like cells were identified in the areas with desmoplastic reactions (Figure 2B). A similar observation was made in all the secondary/metastatic tumor tissues (Figure 2B; data not shown). Additionally, the toluidine blue staining revealed the presence of mast cells (MCs) in different zones of the primary tumors (Figure 2C), as reported in human PC tissues [20]). Multiple MCs were present close to the blood vessels; their number was increased in areas with a greater number of blood vessels. These observations also corroborate a previous report [20].

### Isolation, culture, and characterisation of hamster normal PSCs

**Figure 3:**
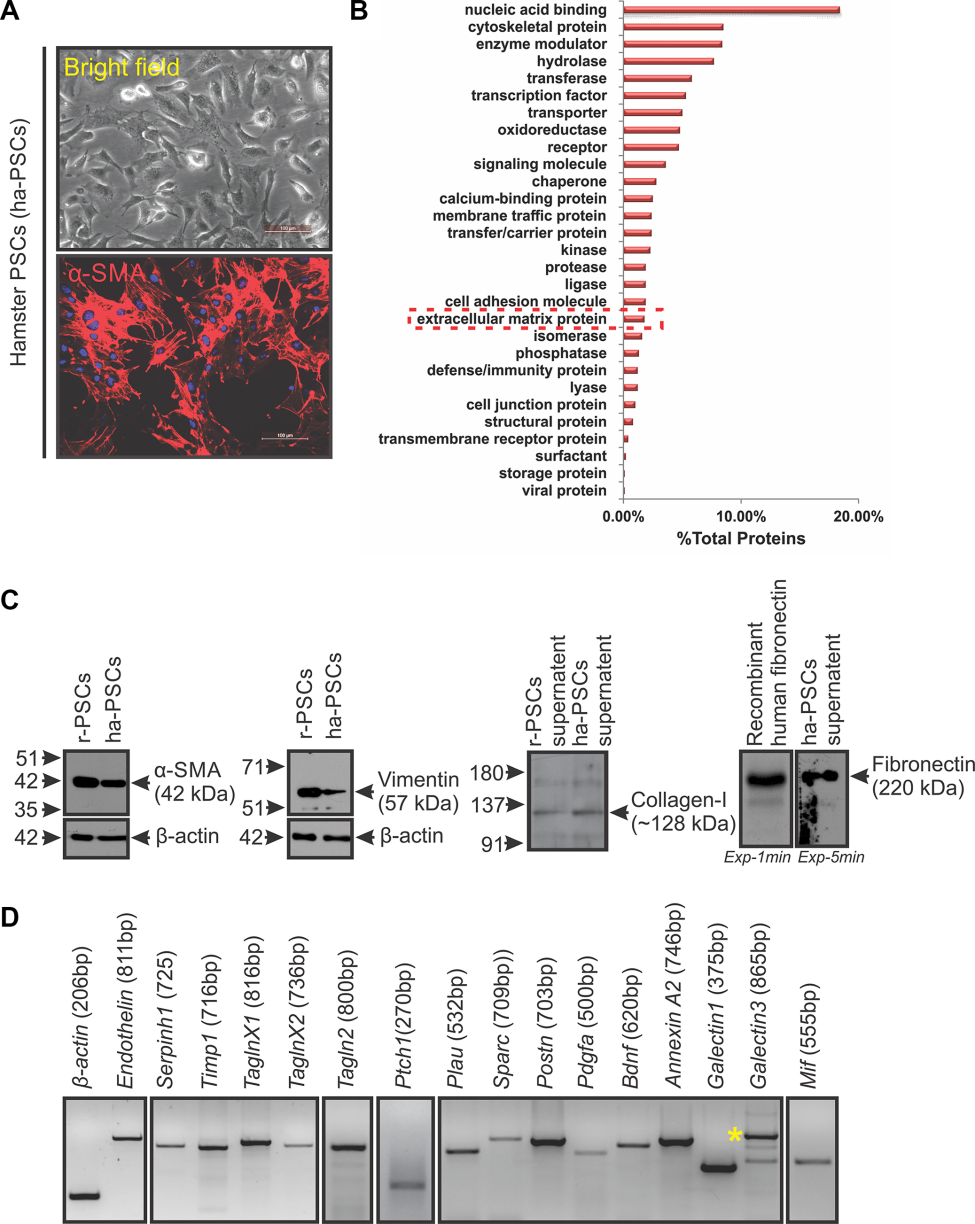
Isolation and molecular characterization of hamster PSCs (ha-PSCs) (**A**) Bright field image of ha-PSCs after 2nd passage showing star-shaped cells with clear cytoskeletal structures (typical morphology of activated PSCs). Immunofluorescence staining for α-SMA showed a homogenous population of activated PSCs (scale bar = 100 μm). (**B**) Mass spectrometric analysis of proteins obtained from in-culture activated ha-PSCs and their further GOPC analysis via the PANTHER classification system shows presence of various types/classes of proteins, including multiple extracellular matrix proteins. (**C**) Immunoblot analysis for certain PSCs-associated proteins (α-SMA, vimentin, collagen I and fibronectin) shows cross-reactivity of different commercially available antibodies against hamster proteins and confirms expression of these proteins by ha-PSCs. Protein lysates obtained from rat PSCs (r-PSCs) and recombinant human-fibronectin were used as positive controls. β-actin was used as loading control. Exp-1min and Exp-5min indicates exposure of the same membrane to two different films for 1 minute and 5 minutes, respectively. (**D**) PCR analysis products for some of the selective molecules confirms expression of these genes in ha-PSCs. β-actin was used as an internal control. Asterisk indicates galectin-3 specific band.

After the second passage of the isolated ha-PSCs, the culture plates had a homogeneous population of cells with a clear morphology of activated PSCs, as reported in other species (Figure 3A) [21]. Further, immunocytochemistry of these cells for α-SMA expression confirmed their purity and activated state (Figure 3A). Activated PSCs are known to express various proteins that directly and/or indirectly contribute to cancer-associated fibrosis and growth or metastasis of cancer cells [4]. In turn, this study investigated the global protein expression pattern of the isolated, in-culture activated hamster PSCs (ha-PSCs) and inspected them for the expression of different fibrosis and pro-tumorigenic proteins using mass spectrometry. Because the Syrian hamster (*Mesocricetus auratus*) genome is not yet fully sequenced, this study used an in-house modified UniProt for human, mouse, and hamster proteins and the National Center for Biotechnology Information (NCBI) hamster database, along with the common contaminant protein sequences for the identification of proteins. A total number of 1,878 proteins were identified (Supplementary Table S3). Further, gene ontology protein classification (GOPC) analysis of the identified proteins showed the expression of different molecules involved in multiple cellular and molecular processes (Figure 3B, Supplementary Table S3). A selective search of the identified protein list showed expression of multiple activated-PSCs markers (*i.e.*, α-SMA, vimentin, desmin and nestin), extracellular matrix proteins (*i.e.*, collagen, fibronectin and laminin), and extracellular matrix remodelling proteins (*i.e.*, matrix metalloproteases and secreted protein acidic and rich in cysteine) [22, 23]. The expression of proteins transgelin and galectin-1 was also found; these proteins are reported to be expressed by activated PSCs and to regulate the function of PSCs and/or the progression of PC [24, 25]. In addition to the proteins that are known to be expressed by PSCs, results showed the expression of certain other proteins such as macrophage migration inhibitory factors (MIFs) and galectin-3 (Supplementary Table S3; Figure 3D), which are known to promote the progression of PC but have not been reported to be expressed by PSCs [26, 27]. The present study also validated expression of certain selected molecules (Figure 3C and 3D) using immunoblot analysis and PCR, followed by sequencing. Given that this study identified 1,878 proteins in triplicate runs of unfractionated protein lysate, some of the previously reported proteins known to be expressed by activated human/rodent PSCs were absent in our dataset. This may be due to low abundance of these proteins in the lysate. To ascertain the expression of missing proteins by ha-PSCs, namely *Endothelin*, *Plau*, *Postn*, *Ptch1*, *Timp1*, and *Pdgfa*, PCR was performed, followed by sequencing of amplified products to determine their expression status (Figure 3D). Together, this expression analysis showed similarities between ha-PSCs and human PSCs [22, 28–30].

### Pirfenidone inhibits HapT1 tumor growth and desmoplastic reactions, and NAC enhances these inhibitory effects of pirfenidone

**Figure 4:**
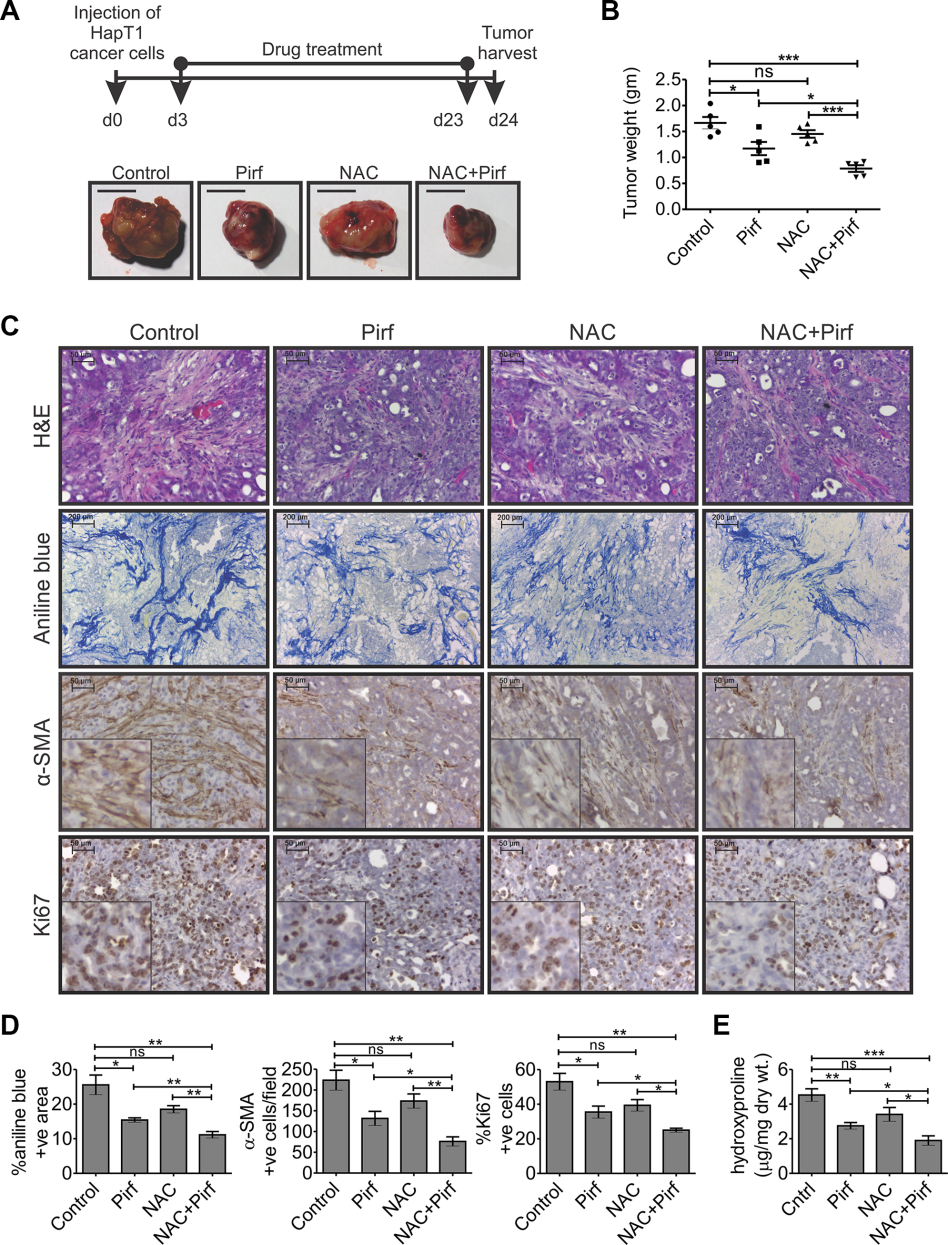
Effects of pirfenidone and/or N-acetylcysteine (NAC) on the HapT1 orthotopic tumor (**A**) Hamsters orthotopically injected with 8 × 10^4^ HapT1 PCCs were orally administered with pirfenidone (Pirf) and/or N-acetylcysteine (NAC) daily for 21 days. The schematic diagram shows the drug treatment schedule for different groups (*n* = 5 animals/group). Representative images of tumors harvested from different groups show smaller tumors in all the drug-treated animals compared with control animals. (**B**) Quantification of tumor weights shows reduction in the tumor weight of all the drug-treated animals compared with the control animals. Pirfenidone and NAC co-administration showed a maximum reduction in tumor weight compared with the individual treatments. (**C**) Histopathological analyses through H&E staining, aniline blue staining, and IHC for α-SMA show a decrease in the extent of desmoplastic reaction in all the drug-treated animals. IHC for Ki67 shows the proliferative cells present in different tumor tissues. (**D**) Quantification of aniline blue, α-SMA, and Ki67 stained sections shows the relative reduction in collagen deposition, the number of activated PSCs/myofibroblast-like cells, and the number of proliferative cells in all the drug-treated tumors compared with control groups, respectively (*n* = 5; each value presents the average of two stained sections per tumor). (**E**) Level of hydroxyproline reflecting collagen content in tumor tissues obtained from different groups of animals (*n* = 5/group). Data presented as mean ± SE; *p < 0.05; **p < 0.005; ***p < 0.0005.

To investigate the effects of pirfenidone and/or NAC in an immunocompetent desmoplastic PC model, HapT1 tumor-bearing hamsters were treated with pirfenidone and/or NAC. Throughout the experiment, there were no visible stress signs or significant changes in the body weight of the individual animals (data not shown). Interestingly, after 21 days of drug treatment, the animals treated with both the drugs in combination had significantly decreased tumor weights than those of animals treated with pirfenidone alone (*p* = 0.0426; Figure 4A, 4B) or only NAC (*p* = 0.0002; Figure 4A, 4B) or only vehicle/control (*p* = 0.0005; Figure 4A, 4B). Only pirfenidone-treated animals had significantly smaller tumors compared with controls (*p* = 0.0233 Figure 4A, 4B); however, only NAC treatment alone did not significantly alter the tumor weight compared to control groups (Figure 4B). Further, histopathological and biochemical analysis showed that pirfenidone in combination with NAC significantly reduced the number of α-SMA-positive cells and collagen deposition compared to controls or only pirfenidone or only NAC-treated animals (Figure 4C–4E). Quantification of Ki67-positive cells showed a significant reduction in number of proliferating cells in animals treated with both the drugs together compared to pirfenidone alone (*p* = 0.0457) or only NAC treated individuals (*p* = 0.0153; Figure 4C, 4D).

### DSF and DSF-Cu have direct cytotoxic effects on PSCs and HapT1 cells; their cytotoxic effect on hamster PSCs and HapT-1 cells is ROS-dependent and -independent, respectively

**Figure 5:**
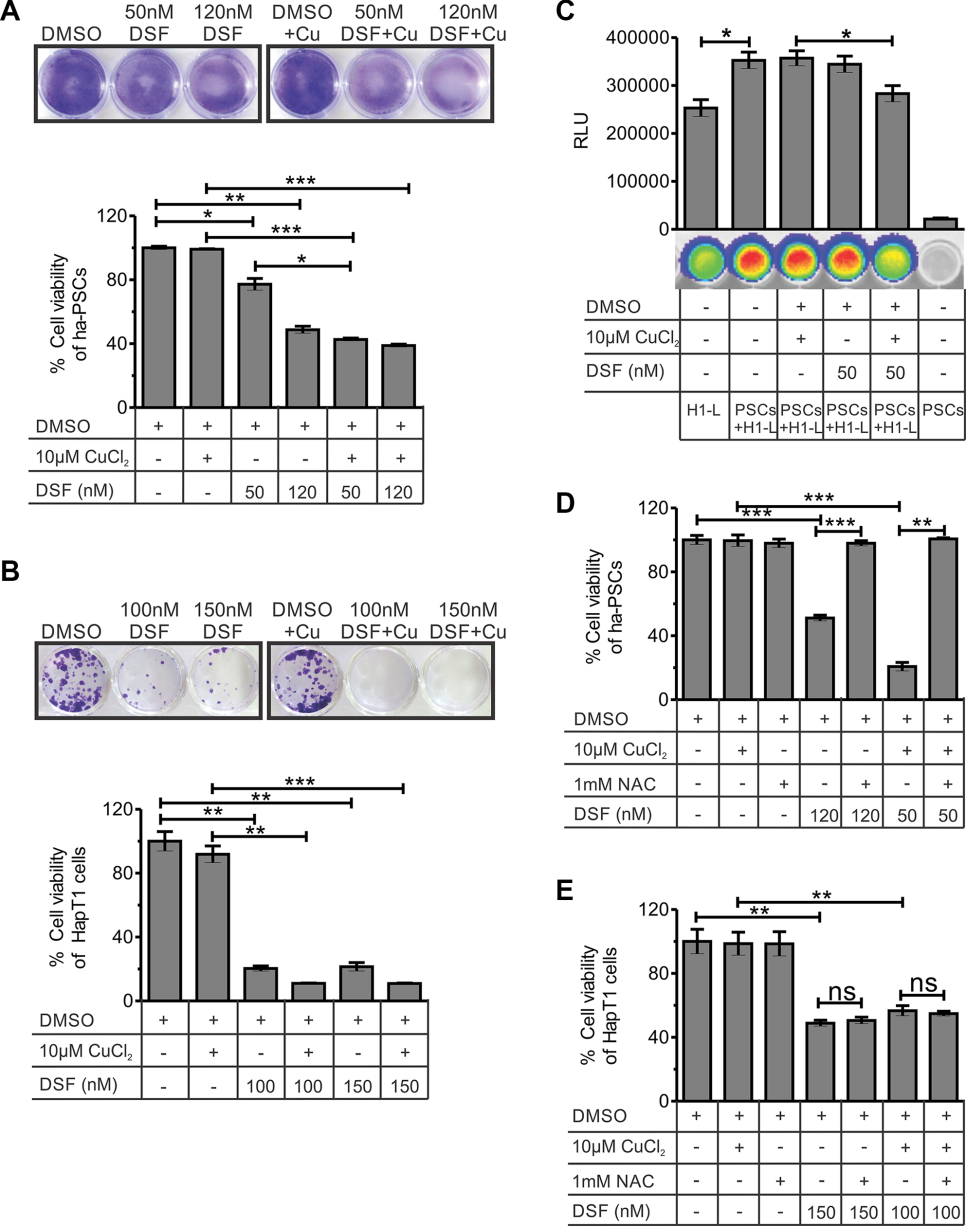
Effect of DSF and/or CuCl^2^ on the viability of hamster-PSCs and HapT1 cancer cells (**A**) After 24 hours of treatment with DSF or DSF+Cu or vehicle control, the ha-PSCs were cultured with normal culture medium. On fifth day of culture, the plates were processed for crystal violate staining and quantification. The data clearly show the significant cytotoxic effect of 120 nM DSF (with and without 10 μM CuCl^2^) and 50 nM DSF + 10 μM CuCl^2^ on ha-PSCs. The figure shows representative images of the crystal violate stained plates (one out of three independent experiments in duplicates) and its corresponding quantified values (*n* = 3). (**B**) The colony forming assay using HapT1 cancer cells shows the cytotoxic effect of DSF alone and in combination with Cu (24 hours of treatment followed by 5 days of culture). Representative image of a crystal violet-stained plate (one out of three independent experiments in duplicates) is shown. Data obtained from the crystal violate quantification corresponds to the number of viable cells (*n* = 3). (**C**) Co-culture of ha-PSCs and HapT1-Luc cancer cells (H1-L) shows the role of PSCs in the growth of cancer cells. However, pre-treatment of PSCs with DSF+Cu significantly reduced the growth advantage conferred by PSCs to H1-L cells. D & E) Cell viability checked through MTT assay showed the rescue of ha-PSCs form DSF-mediated cell death after pre-incubation with 1 mM NAC; however, HapT1 cells did not get benefit from NAC pre-incubation. Data presented as mean ± SE; *p < 0.05; **p < 0.005; ***p < 0.0005.

In an effort to identify novel drug candidates for PC desmoplasia, a FDA-approved drug library screening was performed against the survival of activated rat-PSCs in culture (Supplementary Figure S2A). Out of the 18 drugs selected (data not shown), initially one candidate, DSF, was chosen and validated against PSCs of different origins (Figure 5A; Supplementary Figure S2B and Supplementary Figure S3A). After 24 hours of drug treatment, followed by five days of culture, a significant reduction in cell viability of PSCs was observed when using 120 nM DSF (except human) and 50 nM DSF with 10 μM CuCl_2_ combination for all types of PSCs. The cells treated with only dimethyl sulfoxide (DMSO) or DMSO with 10 μM CuCl_2_ showed no significant effect on the viability of PSCs (Figure 5A and Supplementary Figure S3A).

Both MTT and colony formation assay showed cytotoxic effects for DSF or DSF-Cu in HapT1 cancer cells (Figure 5B and Supplementary Figure S3B). In co-culture models, PSCs are known to promote growth, chemo-resistance, and radiation resistance in PCCs [31]. Based on the above data, we hypothesized that cytotoxic effect of DSF on PSCs will suppress the growth advantage provided by these cells to cancer cells *in vitro*. To check this hypothesis, we established a co-culture system of ha-PSCs and ha-PCCs (HapT1) in which the HapT1cells are labelled with a luciferase gene. The data shown in Figure 5C clearly suggest that ha-PSCs indeed promote growth of HapT1 PCCs. Importantly, pre-treatment of PSCs with 50 nM DSF and 10 μM CuCl_2_ for 12 hours only significantly reduced the growth promoting effect of PSCs on HapT1 cells (*p* = 0.0484). However, within this time frame, 50 nM DSF alone was not sufficient to reduce the growth advantage provided by PSCs to HapT1 cells. In a parallel experiment, only PSCs were cultured and treated with 50 nM DSF or 50 nM DSF with 10 μM CuCl_2_ for 12 hours and processed for crystal violet staining. The data obtained from this experiment showed reduced numbers of PSCs after 12 hours of treatment with DSF and Cu; however, in other conditions there was no significant change (data not shown). Together, the results suggest that the DSF/DSF-Cu-induced reduction in the number of PSCs and/or change in their secretory properties is responsible for reducing the growth promoting effects conferred by PSCs to PCCs. DSF is known to induce cell death in multiple cancer cells by enhancing reactive oxygen species (ROS) [32]. Therefore, to determine whether the effect of DSF-mediated cell death is through ROS, this study examined the viability of ha-PSCs and HapT1 cancer cells that were pre-treated with n-acetylcysteine (NAC) at a 1 mM concentration. Analyses of the data presented in Figure 5D and 5E and Supplementary Figure S2C clearly show the suppression of DSF or DSF-Cu-mediated cytotoxicity in rat and hamster PSCs by NAC; however, NAC pre-treatment failed to supress cytotoxic effect of DSF on HapT1 cancer cells.

### DSF alone partially suppressed the growth of HapT1 orthotopic tumors in a syngeneic host but did not suppress desmoplastic reactions

**Figure 6:**
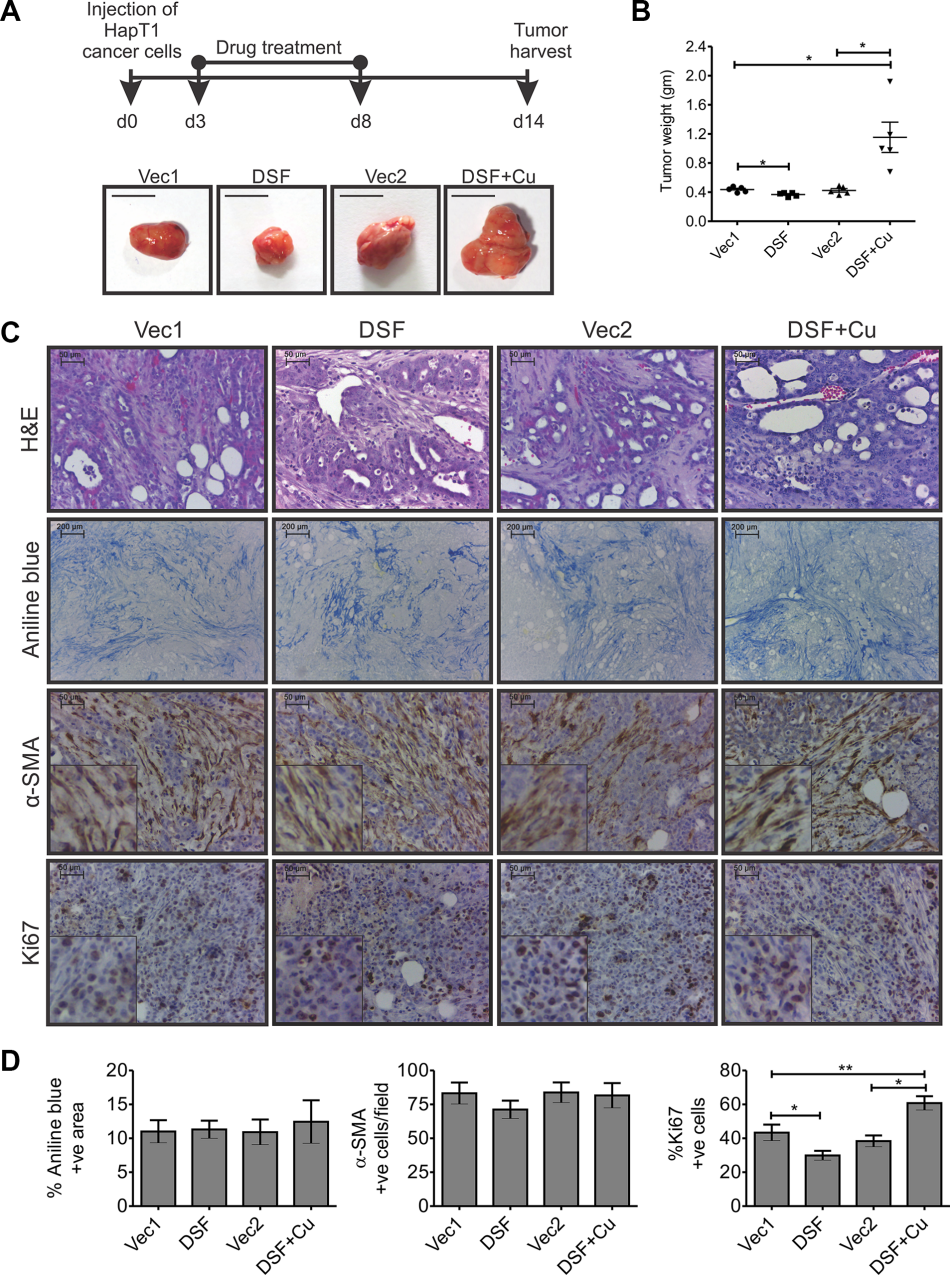
Effect of DSF, with or without Cu, on HapT1 orthotopic tumors (**A**) Hamsters orthotopically injected with 8 × 10^4^ HapT1 PCCs were injected (i.p.) with DSF, or DSF+Cu, or the respective vehicle control. The schematic diagram shows the treatment schedule for different groups (*n* = 5). Representative images of tumors harvested from different groups and quantification of tumor weights show smaller tumors in only DSF-treated animals compared with vehicle controls. However, animals treated with DSF+Cu have larger tumors compared with control animals. (**B**) Quantification of tumor weights shows a significant reduction in the tumor weight only in DSF-treated tumors compared with corresponding control tumors. (**C**) Histopathological analysis through H&E staining, aniline blue staining, and IHC for α-SMA shows similar levels of desmoplastic reaction in all the primary tumors, irrespective of the type of treatment. IHC for Ki67 shows the proliferative cells present in different tumor tissues. (**D**) Quantification of aniline blue and α-SMA stained sections shows similar levels of collagen deposition and abundance of activated-PSCs/myofibroblast-like cells in all groups. Quantification of Ki67-stained sections shows the relative number of proliferative cells in all the drug-treated tumors compared with their corresponding control groups (*n* = 5; each value presents the average of two stained sections per tumor). Data presented as mean ± SE; *p < 0.05; **p < 0.005.

Although the anticancer activity of DSF or DSF-Cu against PCC lines has been established previously [15– 18], its effect on cancer-associated PSCs and/or on cancer-associated fibrosis has remained unclear. Importantly, the effect of DSF on the growth of desmoplastic tumors has not yet been addressed. Therefore, the present study aimed to determine the effect of DSF on a HapT1 desmoplastic tumor model. At the end-point evaluation, we were confounded to see that animals injected with DSF-Cu demonstrated a greater tumor burden (both primary and metastatic) compared with any other group (Figure 6A; data not shown). Animals injected with only DSF showed, on average, a 16% reduction in primary tumor weight and decreased metastasis (data not shown) compared with the corresponding animals treated with the vehicle (Figure 6A and 6B). Between the two vehicle treatment groups, there was no significant difference in the primary tumor weights. Further, the H&E stained sections showed desmoplastic reactions in all the groups (Figure 6C). Moreover, estimation of collagen deposition (through aniline blue staining) and the number of α-SMA positive cells showed no significant difference among animals of various groups (Figure 6C and 6D). Finally, quantification of Ki-67 positive cells showed a significant decrease and increase in the number of proliferative cells in DSF and DSF+Cu treated groups compared with their corresponding controls, respectively (Figure 6C, 6D).

## DISCUSSION

Currently, a significant proportion of studies in human PC employ the orthotopic implantation of PCCs in athymic mice. However, there is experimental evidence that syngeneic tumors are clearly more reliable compared with those developed in athymic mice [33]. In our current study, the presence of extracellular matrix proteins (*e.g.*, collagen and fibronectin) and stromal cells (*e.g.*, myofibroblast-like and mast cells) in HapT1 tumors mimics the desmoplastic reactions seen in human PDAC (Figure 2). Moreover, the presence of similar types of extracellular matrix proteins and their extent of deposition in metastatic tumors corroborates findings in human PDAC patients [19]. In syngeneic experimental tumors like the HapT1 orthotopic tumor, due to the functional immune system of the host, the inflammatory milieu in tumor microenvironment is more robust compared with tumors grown in immune-compromised hosts. Moreover, this could be the major cause behind enrichment of a large number of myofibroblast-like cells and immune cells in HapT1 tumors. In HapT1 tumor tissues, the presence of Ki67-positive stromal or myofibroblast-like cells indicates a certain level of proliferative activity of these cells (Supplementary Figure S4). Hence, recruitment of myofibroblast-like cells and/or their further proliferation in tumor microenvironment might have contributed to the overall population of these cells.

Accumulating evidence suggests that mast cells promote the progression of PC [20, 34, 35]. In a 2015 study, Masso-Valles D *et al.* showed that Ibrutinib, a mast cell inhibitor, restricted fibrosis associated with PDAC and extended the survival time of animals bearing these tumors [36]. Tumor tissue derived from the HapT1 cells contained numerous mast cells (Figure 2C). Whether this could be employed in the future to investigate the effect of mast cell inhibitors on PC fibrosis and growth remains an unanswered question.

This study also describes the process of isolation and characterization of ha-PSCs (Figure 3A–3D). The protein expression profile of ha-PSCs was similar to that of rat and human suggesting that similarities exist between PSCs from different species. A robust expression of Sonic hedgehog (Shh) was noted in HapT1 cells (Supplementary Figure S5A and S5C) and that of *Ptch1* in hamster PSCs (Figure 3D). This finding is important as binding of SHH produced by PCCs with its receptor PTCH1 expressed by PSCs activates PSCs proliferation, desmoplasia, and growth of PCCs [37]. N-terminal SHH is known to promote desmoplasia in human PC. We found that human and hamster N-SHH are 99% identical (Supplementary Figure S5B). This supports the usefulness of this hamster model to test novel therapeutics against human PC. Identification of MIF expression in activated ha-PSCs, r-PSCs, and hu-PSCs (Supplementary Table S3; Figure 3D; data not sown) has opened a novel aspect in PSCs-mediated PC progression. A 2015 study found that MIF is highly expressed in PDAC-derived exosomes, and primes the liver for metastasis [27]. Ongoing studies are attempting to understand the effect of PSCs-derived MIF on the properties of PSCs and PCCs.

Pirfinidone treatment significantly decreased growth of orthotopically implanted HapT1 tumors compared to controls. It also decreased intra-tumor collagen deposition, and the number of α-SMA positive cells (Figure 4). These findings corroborate previous reports of the anticancer and antifibrotic property of pirfenidone [11]. A combination of pirfenidone and NAC decreased tumor growth more than pirfenidone or NAC alone suggesting a possible therapeutic use of this combination in treating human PC. Pirfenidone and NAC are known to reduce fibrosis by inhibiting TGF-β and ROS in fibrogenic cells, respectively [38, 39]; however, the exact mechanism behind their combinatorial effect against PSCs and PCCs is yet to be investigated.

In the current study, we observed a significant cytotoxic effect of DSF or DSF+Cu against in-culture activated ha-PSCs and HapT1 cancer cells (Figure 5A and 5B); however, the failure of DSF to perform in a physiologically relevant HapT1 orthotopic tumor animal model (Figure 6) strongly suggests the importance of using appropriate animal models in preclinical drug validation. The *in vitro* cytotoxic effects of DSF on ha-PSCs and HapT1 cancer cells were ROS-dependent and -independent, respectively (Figures 5D and 5E). The origin of cancer-associated fibroblast cells (CAFs) is potentially from different sources and constitutes a heterogeneous population [6]. Therefore, to rule out the possibility that DSF is ineffective against cancer-associated fibroblast cells, we isolated these cells from HapT1 orthotopic tumor tissues and treated them with DSF or DSF+Cu (Supplementary Figure S6). Like PSCs, the cancer-associated fibroblast cells were susceptible to DSF or DSF+Cu treatment, and the cytotoxic effect of DSF was ROS-dependent *in vitro*. Studies by others have shown that the initial level of ROS in cells is critical for DSF-induced cell death [32, 40]. By the same token, it is a well-established fact that many cells isolated from normal tissues and maintained in culture have increased levels of culture-induced ROS [41]. However, such increased basal levels of ROS are rarely or never been seen in any normal cells of humans or animals *in vivo* [41]. Hence, agents like DSF that induce cell death through enhancing ROS beyond a tolerable level potentially do not show their cytotoxic effect *in vivo*. A moderate cytotoxic effect of DSF on cancer cells, as evidenced by a 16% reduction in tumor weight, is potentially due to the ROS-independent cytotoxic effect of DSF on HapT1 cancer cells (Figure 6B). It is possible that the failure of DSF alone to induce a highly significant antitumor effect *in vivo* is due to decreased bioavailability. The failure of DSF+Cu to reduce both desmoplastic reactions and overall tumor burden can be viewed as a harbinger for the unwanted pro-tumorigenic events that could occur due to the Cu-induced hyperactivity of DSF. In past decade, a Cu and DSF combinatorial approach has shown encouraging results in immune-compromised tumor models [15–18]. However, based on the known protumorigenic effect of Cu alone [42] and our current observation, we expect exogenous Cu would induce toxicity in DSF-treated animals. Together, our investigation reinforces the importance of using a physiologically relevant animal model, like the HapT1 hamster tumor model, for validating the efficacies of drugs that are more likely to be effective in humans.

## MATERIALS AND METHODS

### Orthotopic injection of HapT1 PCCs and drug administration

Prior to conducting any animal studies, all protocols were approved by the Institutional Animal Ethical Committee (Institute of Life Sciences, Bhubaneswar, India). To develop the desmoplastic pancreatic tumor, HapT1 PCCs (8 × 10^4^) were orthotopically injected into the pancreases of Syrian golden hamsters. For this purpose, inbred Syrian golden hamsters were procured from the National Institute of Nutrition in Hyderabad, India. HapT1 cells were harvested from sub-confluent culture by a brief exposure to 0.25% trypsin and 0.02% ethylenediaminetetraacetic acid (EDTA). After neutralising trypsin with 10% fetal bovine serum (FBS), the cells were washed and re-suspended in phosphate-buffered saline (PBS) at a concentration of 8 × 10^4^ cells/50 μl. Single-cell suspensions of greater than 90% viability were used for injection. Animals were anesthetized with intraperitoneal (i.p.) administration of a cocktail mixture of ketamine (150 mg/kg) and xylazine (10 mg/kg). After sterilizing the incision site, an approximately 1cm long incision was made near the position of spleen. Gently, the splenic lobe of the pancreas (Figure 1A) along with the spleen was pulled up to the incision site, and around 8 × 10^4^ HapT1 cells in 50 μl volume were injected into the splenic lobe of the pancreas using a 30-guage needle. After resetting all the pre-moved organs, the abdomen was closed with absorbable catgut sutures. The skin incision was closed with interrupted sutures of non-absorbable material. The skin suture material was removed at approximately five days after the surgical procedure. The animals were monitored daily.

To analyse the effects of pirfenidone or NAC alone or the combination of pirfenidone + NAC, HapT1 (8 × 10^4^) cells were orthotopically implanted into the splenic lobe of the hamster pancreas (Figure 1A). Pirfenidone was purchased as Pirfenex tablets (Cipla Ltd), crushed using a mortar and pestle, and dissolved in sterile water to a pirfenidone concentration of 200 mg/ml. NAC (Sigma) was dissolved in sterile drinking water at a concentration of 1 g/L. Three days after the injection of cancer cells, all the tumor-bearing animals were randomly divided into four groups (*n* = 5 per group) and orally administered either 500 mg/kg pirfenidone (once daily) and/or 1 g/L NAC in drinking water for 21 continuous days. Control animals were provided with only sterile drinking water. At day 24, all the animals were sacrificed, tumor weights were measured, and gross necropsy was completed to determine the extent of metastasis.

To evaluate the effect of DSF and DSF+Cu, HapT1 (8 × 10^4^) cells were orthotopically implanted into the pancreases of hamsters. Three days after injecting cancer cells, all the tumor-bearing animals were randomly divided into four groups (*n* = 5 per group). Before the injection into animals, DSF (MP Biomedical) was dissolved in a solution of DMSO, olive oil, cremophor, and PBS (0.5:3:1.5:5). Each group received 200 μl i.p injection of the following treatments for six days: (i) vehicle1 group (Vec 1): DMSO-olive oil-cremophor-PBS, (ii) DSF group: DSF 50 mg/kg, (iii) vehicle2 group (Vec 2): 0.5 mg/kg CuSO_4_ in DMSO-olive oil-cremophor-PBS, and (iv) DSF+Cu group: DSF 50 mg/ kg and 0.5 mg/ kg CuSO_4_. At day 14, all animals were sacrificed, tumor weights measured, and gross necropsy was performed to determine the extent of metastasis.

### Tissue processing and haematoxylin and eosin staining

Tissue samples were collected and preserved in 10% formalin buffer solution at room temperature. For histopathological analysis, tissues were processed for paraffin embedding, and multiple 5-micron sections were prepared. For haematoxylin and eosin staining, slides were deparaffinized and hydrated with deionized water followed by haematoxylin (Sigma) staining for three minutes and eosin for two minutes. Next, slides were thoroughly washed in H_2_O and dehydrated through sequential alcohol grading then cleared in xylene and mounted with permanent mounting media (Vector Lab). Stained slides were observed under a Leica DM500 light microscope, and representative images were taken at 10× and 40× magnifications.

### Aniline blue staining

For aniline blue staining, we modified the conventional trichrome staining protocol. Briefly, slides were deparaffinized and hydrated with deionized H_2_O followed by incubation in pre-heated Bouin's solution (Sigma) for 15 minutes. Next, slides were cooled at room temperature and washed under running tap water. Tissue sections were then incubated in working phosphotungstic-phosphomolybdic acid solution (Sigma) for five minutes followed by aniline blue (Sigma) for 10 minutes and rinsed in 1% acetic acid (SRL) for 2 minutes. Finally, slides were thoroughly washed with deionized H_2_O and dehydrated through sequential alcohol grading. They were then cleared in xylene and mounted with permanent mounting media (Vector Lab). Stained slides were observed under a Leica DM500 light microscope, and images were taken at 10× and 40× magnifications. The percentage of collagen stained area (aniline blue) was quantified using the automated ImageJ program in conjunction with the threshold plug-in [43].

### Toluidine blue staining

For toluidine blue staining, briefly, the slides were deparaffinized and hydrated with deionized H_2_O, followed by staining with toluidine blue solution pH 2.3 (Merck) for three minutes. They were subsequently washed with deionized H_2_O three times. Slides were dehydrated quickly using 95% and 100% ethanol and then cleared in xylene and mounted with permanent mounting media (Vector Lab). Stained slides were observed under a Leica DM500 light microscope, and images were taken at 10× and 40×magnifications.

### Immunohistochemistry

For immunohistochemistry (IHC), mouse monoclonal anti-αSMA, rabbit monoclonal anti-vimentin, and rabbit polyclonal anti-fibronectin primary antibodies were used to detect the cellular and extracellular proteins (Supplementary Table S1). Briefly, slides were deparaffinized and hydrated with deionized water. Antigen retrieval was perfomed in acidic pH citrate buffer (Vector Lab) by incubating slides in a steam cooker for 20 minutes. Slides were then washed twice with PBS for five minutes, followed by endogenous peroxidase quenching in 3% H_2_O_2_ (SRL) for 15 minutes. Nonspecific binding was blocked by incubating the slides with horse serum for 30 minutes, followed by overnight incubation with primary antibody α-SMA, vimentin, and fibronectin at 4^°^C in a humidified chamber. Details of antibody dilutions are provided in Supplementary Table S1. Slides were then washed twice with PBS for five minutes and incubated with biotinylated anti-rabbit/mouse IgG secondary antibody for 45 minutes. Diaminobenzidine (Vector Lab) was used to detect the immunoreactivity. Slides were subsequently counterstained with haematoxylin (Sigma), dehydrated through sequential alcohol grading, cleared in xylene, and mounted with permanent mounting media (Vector Lab). Stained slides were observed under a Leica DM500 light microscope, and images were taken at 10× and 40× magnifications. For all type of histopathological evaluations, the stained slides were assessed by a qualified veterinary pathologist (SKP).

### Hydroxyproline assay

Frozen HapT1 tumor tissues were homogenized using mortar pestle and lyophilized. Further, collagen estimation was carried out as per the instructions provided with the hydroxyproline assay kit (Sigma; MAK008). Briefly, 5 mg of dry tumor samples in 250 μl of 12 M HCl was hydrolyzed at 120°C for 3 hours. The hydrolyzed samples were then briefly centrifuged and 50 μl of supernatant were aliquoted to 96-well plate in duplicate and allowed to dry in a 60°C oven. Next, 100 μl of chloramine T/oxidation buffer mixture was added to each well and incubated at room temperature for 5 minutes followed by addition of 100 μl of diluted 4-(Dimethylamino)benzaldehyde (DMAB) reagent and incubated for 90 minutes at 60°C. Absorbance was measured at 560 nm on Varioskan^™^ Flash Multimode Reader (Thermo Scientific). Collagen was quantified as μg of hydroxyproline per mg dry weight of starting material.

### Isolation of rat and hamster pancreatic stellate cells

Immediately after sacrificing the animal, the pancreas was collected in pre-chilled PBS containing 1× penicillin/streptomycin (Sigma) under a BSL2 laminar hood and processed as described by MV Apte *et al.* [21]. Harvested cells were washed in 0.3% BSA and resuspended in IMDM medium (Pan-Biotech) containing 10% FBS or 5% hamster serum, and grown in a humidified incubator at 37°C.

### Isolation of cancer-associated fibroblasts from HapT1 tumors

Immediately after sacrificing the tumor-bearing animals, the tumor was collected in pre-chilled PBS containing 1× penicillin/streptomycin (Sigma) under a BSL2 laminar hood. For isolation of cancer-associated fibroblasts, tissues were taken only from peripheral region of the tumor avoiding the necrotic tissues. Tissue samples were disaggregated using a fine scalpel and digested with an enzyme cocktail (collagenaseP 1.3 mg/ml and DNase1 0.01 mg/ml) at 37°C in shaking water bath (180 rpm) for 60 minutes, followed by mechanical dissociation. Digested tissue samples were then filtered through a 70 μm cell strainer (BD Falcon), washed with PBS, resuspended in 5% FBS/hamster serum containing Dulbecco's Modified Eagle Medium (DMEM; Pan-Biotech), and grown in a humidified incubator at 37°C. After 30 minutes of cell attachment, the remaining unattached cells were washed off, and fresh medium was added.

### Immunocytochemistry

Hamster-PSCs of P2 passage, cancer-associated fibroblasts of P3 passage, and rat-PSCs of P3 passage were seeded on poly L-lysine-coated glass coverslips (Sigma) in six-well plates. After achieving the minimum required confluence, cells were washed with PBS twice for five minutes per wash, followed by fixation with an ice-cold mixture of methanol and acetone (1:1 ratio) for 10 minutes at −20°C; nonspecific binding was blocked with 2% BSA (MP Biomedical) in PBS for 30 minutes. Cells were then incubated with an anti-α-SMA primary antibody (1:100) overnight in a humidified chamber at 4°C, followed by anti-mouse IgG-ALEXA FLOUR 594 (1:500) for 45 minutes at room temperature. Finally, cells were washed with PBS and mounted with mounting medium containing DAPI (Life Technologies) to stain the nuclei. Stained slides were examined under a Leica DMIL LED microscope, and images were captured at 10× and 20× magnifications.

### Cell culture and viability assay

The HapT1 cancer cell line was commercially procured from Sigma, and human PSCs (hu-PSCs) were procured from Sciencell Research Laboratories. Both the cell lines were propagated and frozen immediately after receipt. The frozen stocks were revived and used within three months. HapT1 cells were maintained in 10% FBS that contained MEM medium (Pan-Biotech) supplemented with 1x NEAA (Life Technologies). Human PSCs were maintained in 2% FBS containing Stellate Cell Medium (SteCM; Sciencell) supplemented with growth factors. With the approval of Institutional Animal Ethics Committee (Institute of Life Sciences, Bhubaneswar, India), rat and hamster PSCs were isolated from normal pancreas. The PSCs were confirmed to have the typical stellate cell morphology and were positive for α-SMA (a marker for activated PSCs). All of the established cell lines were used between 3-6 passages. Rat PSCs were maintained in 10% FBS containing Iscove's Modified Dulbecco's Medium (IMDM; Pan-Biotech), and hamster PSCs were maintained in 5% hamster serum containing IMDM medium (Pan-Biotech). To check cell viability *in vitro* for HapT1 and PSCs, the MTT assay, crystal violet staining, and colony formation assay were performed as reported earlier [44, 45]. To determine the role of ROS-induced cell death, cells were pre-treated with 1 mM NAC for 12 hours before adding DSF or DSF+Cu.

### Co-culture of ha-PSCs with HapT1-Luc cells

For direct co-culture assays, HapT1 cells were stably transfected with luciferase-expressing plasmid *luc2* (pGL4.50 [*luc2*/CMV/hygro]; Promega) using lipofectamine 3000 (Invitrogen). After 24 hours of transfection, cells were cultured in MEM medium containing selective antibiotic of 750 μg/ml Hygromycin B (MP Biomedicals) to obtain hygromycin-resistant HapT1-Luc clones. ha-PSCs were grown as monolayer and treated with DSF (50 nM) with/without CuCl_2_ (10 μM) for 12 hours. After completion of treatment, drug-containing medium was removed. Next, HapT1-Luc cells were seeded on top of the PSCs and grown for 48 hours. To quantify viable HapT1-Luc cells, cell lysis was performed and checked for luciferase activity with Luciferase Assay System (Promega). In this method the intensity of bioluminescence is proportional to number of viable HapT1-Luc cells. The bioluminescence intensity were measured by IVIS Lumina XR imaging system (Caliper Life Sciences) and Varioskan^™^ Flash Multimode Reader (Thermo Scientific). The values obtained through the multimode reader were plotted as a bar graph.

### Western blot analysis

For isolation of proteins, cells were harvested using a radio-immuno precipitation assay (RIPA) cell lysis buffer (20 mM Tris-HCl pH 7.5, 150 mM NaCl, 1 mM Na_2_EDTA, 1 mM EGTA, 1% NP-40, 1% sodium de-oxy-cholate, 2.5 mM sodium pyrophosphate, 1 mM β-glycerophosphate, 1 mM Na_3_VO_4_) supplemented with a protease inhibitor cocktail (MP Biomedicals). Protein samples were passed through an insulin syringe to facilitate the disruption of cell membranes and centrifuged at 14,000 rpm for 30 minutes at 4°C. Supernatants were collected, and protein concentrations were determined by using the Bradford assay (Sigma). Protein lysates were electrophoresed through 6% (for fibronectin), 8% (for collagen) and 10% SDS-polyacrylamide gels (for α-SMA, vimentin and SHH). The resolved proteins were transferred onto poly-vinylidene difluoride membrane (Milipore) and subjected to a standard immune-detection procedure using specific primary antibodies (Supplementary Table S1) and horseradish peroxidase-conjugated secondary antibodies. Protein expression was visualized by electrochemiluminescence (ECL; Bio-Rad).

### RNA Isolation, cDNA synthesis, RT-PCR, and sequencing

Total RNA was isolated from hamster PSCs and HapT1 cancer cells by using an RNeasy Mini Kit (Qiagen) according to manufacturer instructions. First-strand cDNA synthesis was completed using 2 μg of total RNA and a high-capacity cDNA synthesis kit (Applied Biosystems) according to manufacturer instructions. Expression of different genes were determined using RT-PCR. Notably, *β-actin* served as an internal control. Details of the primer sequences are given in Supplementary Table S2. A total of 2 μl of each cDNA sample was used as a template for performing RT-PCR using Taq Polymerase (Invitrogen). The annealing temperature for each primer set is mentioned in Supplementary Table S2. The PCR products were resolved in 2% agarose gel at 60 V, and presence of amplicons were documented using gel documentation system (BioRad). PCR-amplified products for all the genes were sequenced using Sanger sequencing method (Applied Biosystem).

### Protein isolation for massspectrometry analysis

For isolation of protein samples from hamster PSCs, cells were harvested in protein lysis buffer (0.025 M Tris, 0.15 M NaCl, 0.001 M EDTA, 1% NP- 40, 5% glycerol, pH 7.4) then supplemented with a protease and phosphatase inhibitor cocktail (MP Biomedicals) and stored at −80^°^C until further analysis. The homogenate was centrifuged at 14,000 rpm for 30 min at 4^°^C and the supernatant was collected. Protein concentrations were determined by Bradford assay (Sigma). Five hundred micrograms of protein sample was precipitated with 30% TCA at 4^°^C for 16 hr. The sample was subsequently centrifuged at 14,000 g at 4^°^C for 30 minutes; the supernatant was discarded, and pellet was air dried. Next, the protein pellet was suspended in 50 μl of re-suspension buffer (6 M urea). Protein (30 μg) was denatured by incubating at 60^°^C for 1 hour with 2 μl triflouroethanol (TFE) and then reduced by adding a 20 mM final concentration of diothiothretol (DTT); cysteines were alkylated by adding 20 mM final concentration of iodoacetamide (IAA). Next, the samples were digested overnight at 37°C in a final concentration of 2 M urea with 100 mM Tris- HCl, pH 8.5, containing trypsin at an enzyme/substrate ratio of 1:20 (Promega). The reaction was quenched by addition of 90% formic acid to a final concentration of 4%. Digested samples were stored at −80°C until mass spectrometry analysis.

### LC-MS/MS analysis

Nanoflow electrospray ionization tandem mass spectrometry (Nano ESI-MS) analysis was carried out using a Bruker nanoLC system (Nano Advance, BrukerDaltonics) along with a captive spray- Maxis-HD-qTOF mass spectrometer (BrukerDaltonics) with high accuracy and sensitivity. Digested peptides were desalted, concentrated on C18 reverse phase ZipTips, and eluted twice with 20 μL 60% acetonitrile in 0.1% formic acid before vacuum drying at room temperature. Peptides were resuspended in 10 μL 0.1% formic acid, and subjected to nLC-MS/MS. Peptides were separated using 15 cm long analytical columns (Bruker Magic C18AQ, 0.1 × 150 mm, 3 μm particle size and 200 Å pore size) of 135 minute gradient from 5% to 90% acetonitrile in 0.1% formic acid and a flow rate of 400 nL per minute. The mass spectrometer was operated in data-dependent acquisition mode with 3 intense precursor ions. Exclusion of all charges less than 2 and greater than 6 were used, and all unknown charges were kept in rejection mode. A dynamic exclusion of 20 seconds was used. Full mass spectrometry spectra scans were acquired with a scan range from 350 to 2200 m/z.

### Data processing and bioinformatics analysis

Mass spectrometry data were analyzed by using MaxQuant [46, 47] software version 1.5.2.8 and searched through an in-house modified UniProt human, mouse, and hamster database, along with the NCBI hamster database by adding common contaminant protein sequences. A database search was performed in Andromeda [48] that was integrated in a MaxQuant environment. A list of 280 common laboratory protein contaminants included in MaxQuant was also added to the database, as well as reversed target decoy search, which was used for all sequences. For searching, the enzyme specificity was set to trypsin with the maximum number of missed cleavages set to 2. The precursor mass tolerance was set to 0.07 Da for the first search and then to 0.006 Da for the main search. Mass tolerance for fragment ions in collision-induced dissociation (CID) spectra was set to 0.5 Da. The search included variable modifications of protein N-terminal acetylation, methionine oxidation, and carbamidomethylation of cysteines, and was searched as a fixed modification. The maximal number of modifications per peptide was set to 5. The false discovery rate (FDR) for PSM, proteins, and the site decoy fraction was set to 1%. Additionally, time-of-flight mass spectrometry (TOFMS)/MS mass tolerance was set to be 40 ppm. A minimum peptide length of 6 was set, and the ‘peptide requantification’ function was enabled. To validate and transfer identifications across different runs, the ‘match between runs’ option in MaxQuant was enabled with a retention time window of 0.7 minutes and an alignment time window of 20 minutes. Subsequent bioinformatics analysis were performed using Protein Analysis through Evolutionary Relationships (PANTHER) to compare the gene ontology protein class (GOPC). The obtained PANTHER [49] data was further analyzed, and graphs were prepared using Microsoft Excel 2007. The mass spectrometry data has been deposited to the Proteome X- change consortium with the PRIDE partner repository with the database identifier PXD003385 [50].

### Statistical analysis

Comparisons between groups were evaluated using an unpaired *t* test. Differences were considered significant when the *p*-value was < 0.05. Analyses were conducted by using Microsoft Excel 2007 or GraphPad Prism software. Experimental results are expressed as mean ± SE.

## SUPPLEMENTARY MATERIALS FIGURES AND TABLES




