# Supplementary material for: Characterization and use of HapT1-derived homologous tumors as a preclinical model to evaluate therapeutic efficacy of drugs against pancreatic tumor desmoplasia
Source: Oncotarget. 2016 May 31;7(27):41825–42. doi: 10.18632/oncotarget.9729 (PMC5173099; doi:10.18632/oncotarget.9729)
Supplement: Supplementary file 1 [file oncotarget-07-41825-s001.pdf]

Characterization and use of HapT1-derived homologous tumors as a preclinical model to evaluate therapeutic efficacy of drugs against pancreatic tumor desmoplasia

Supplementary Materials

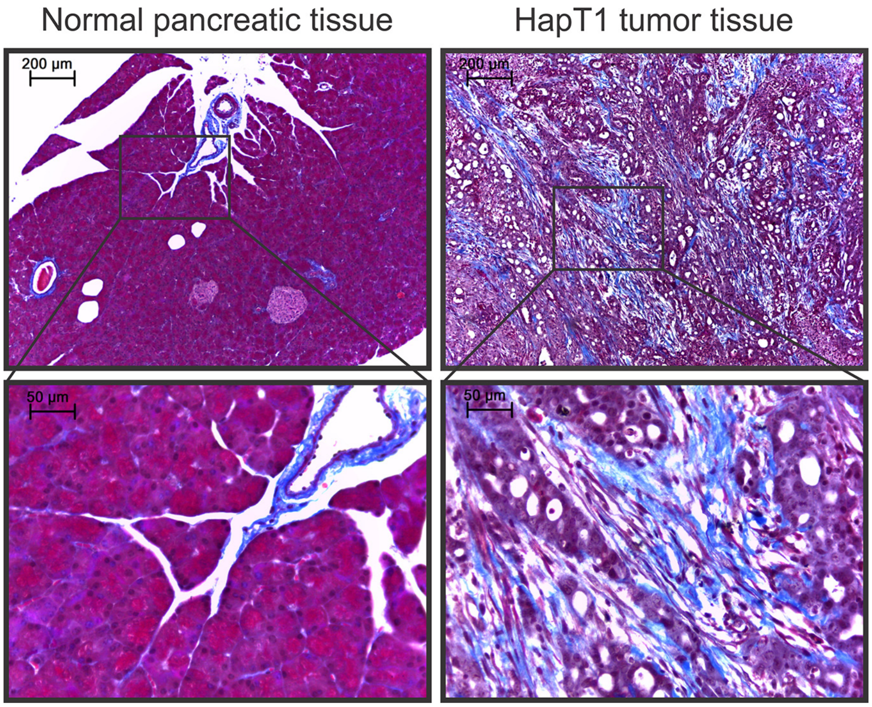

Supplementary Figure S1: Masson's Trichrome staining of HapT1 tumor and normal pancreatic tissue. Masson's Trichrome staining shows the extent of collagen deposition (blue) in HapT1 tumor and normal pancreatic tissue of the same animal. In normal pancreas, blood vessels and ducts have clear collagen staining around them; however, in other parts of the tissue the collagen level is very less or undetectable. On the other hand, HapT1 tumor tissue has an extensive deposition of collagen throughout the tumor parenchyma.

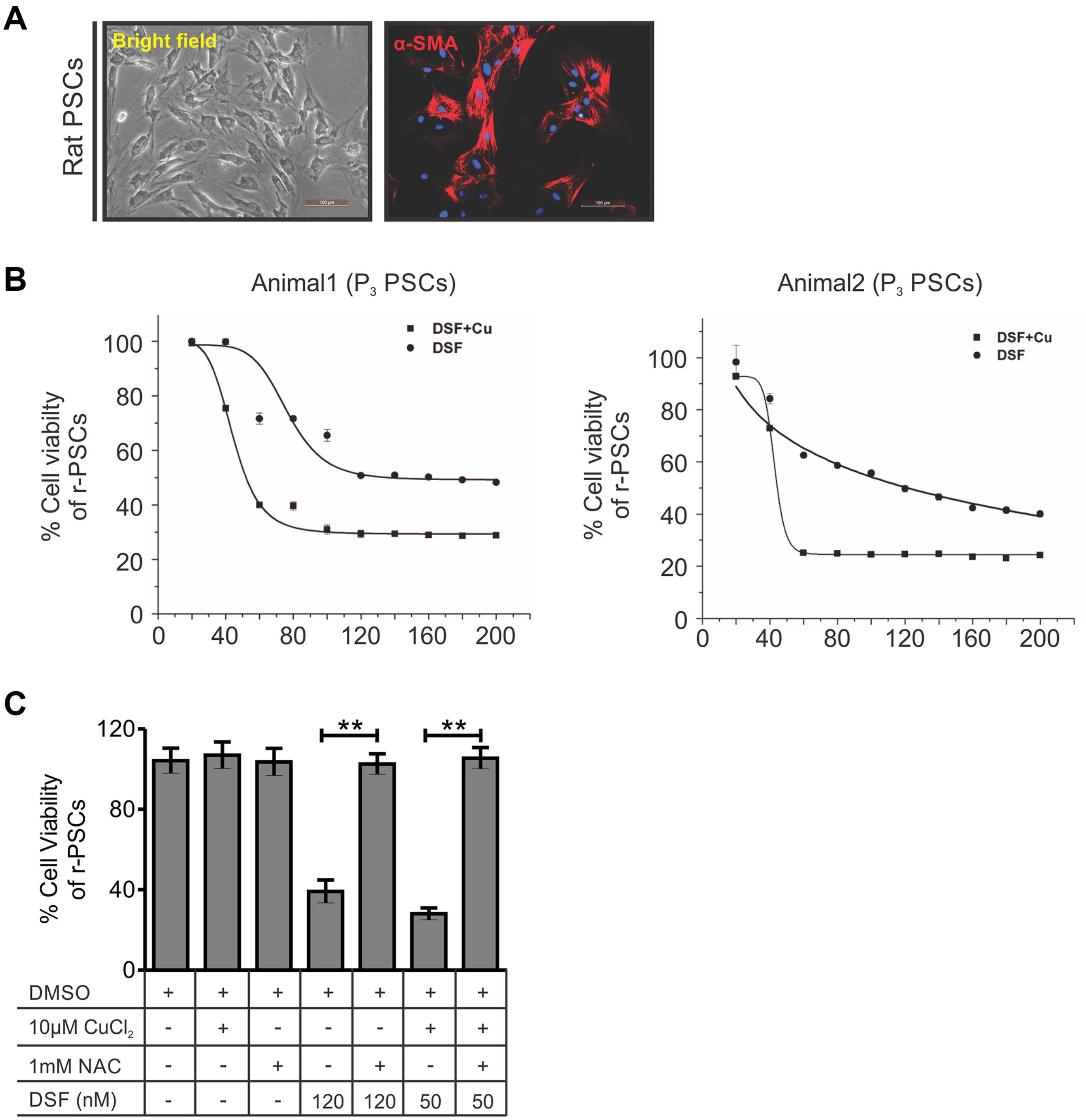

Supplementary Figure S2: Isolation of rat-PSCs (r-PSCs) and evaluation of DSF effect on these cells. (**A**) Bright field image of r-PSCs after 2nd passage shows star shaped cells with clear cytoskeletal structures (typical morphology of activated r-PSCs). Immunofluorescence staining for α-SMA shows homogenous population of activated PSCs. (**B**) Cell viability checked through MTT assay shows the cytotoxic effect of DSF or DSF+Cu on r-PSCs at different concentrations. (**C**) Cell viability checked through MTT assay shows rescue of r-PSCs from DSF-mediated cell death after pre-incubation with 1 mM NAC. The data presented as mean ± SE ($n = 3$) and $^{**}p < 0.005$.

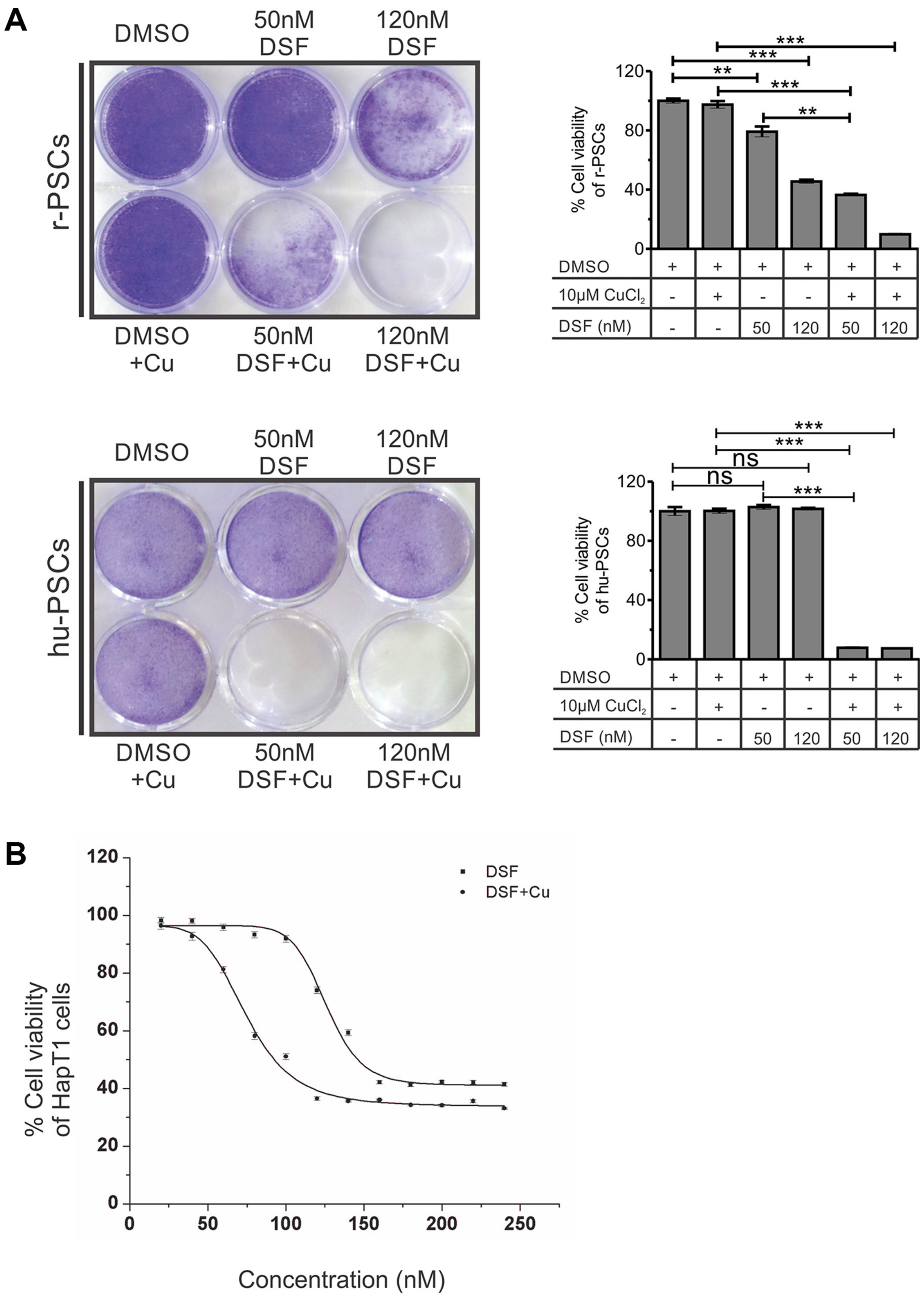

Supplementary Figure S3: Effect of DSF and/or CuCl^2 on the viability of rat-PSCs (r-PSCs), human-PSCs (hu-PSCs) and HapT1 cancer cells. (**A**) After 24-hour treatment with DSF or DSF + Cu or vehicle control, the PSCs were cultured with normal culture medium. On 5th day of culture, the plates were processed for crystal violet staining and quantification. The data clearly shows the significant cytotoxic effect of 120 nM DSF on r-PSCs and 50 nM DSF + 10 µM CuCl^2 on rat and human PSCs. Representative images of the crystal violate stained plates (one out of three independent experiments in duplicates) are shown in left, and quantified values ($n = 3$) are shown in right. The data presented as mean ± SE; $^{**}p < 0.005$; $^{***}p < 0.0005$. (**B**) Direct effect of DSF in presence and/or absence of Cu (48 hr treatment) on HapT1 cancer cells was checked through conventional MTT assay. The data presented as mean ± SE.

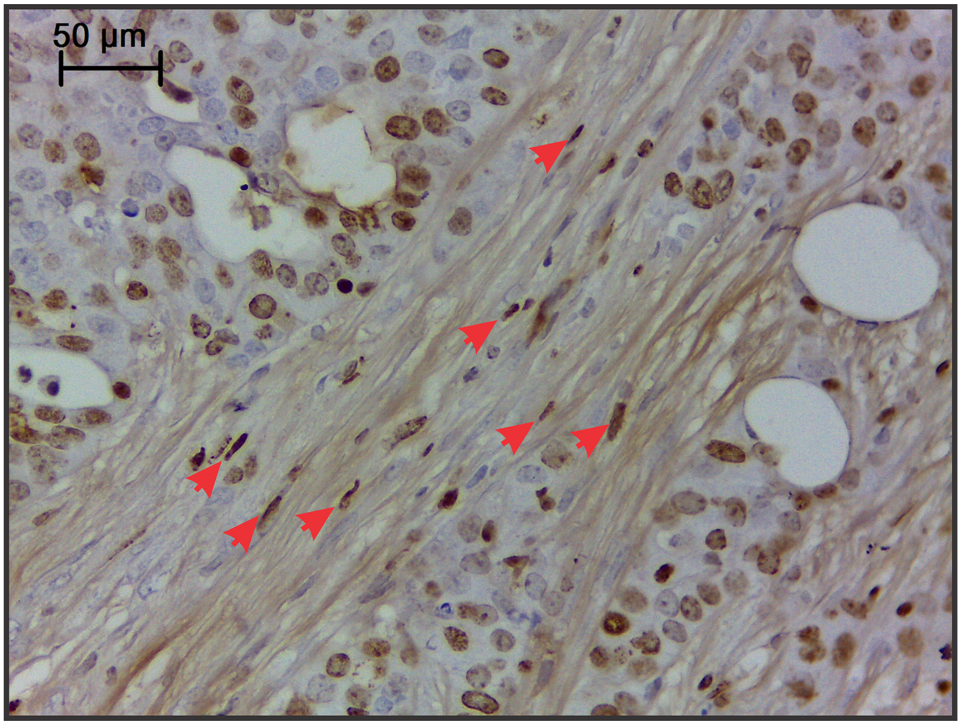

Supplementary Figure S4: Proliferative nature of HapT1 tumor stromal cells *in vivo*. Primary HapT1 tumor tissue sections were stained with a human Ki67 specific antibody. Analysis of stained slides showed cross reactivity of this antibody with hamster protein. Most of the cancer cells (with round and bigger nucleus) are positive for Ki67 (typical nuclear staining); and some stromal cells (spindle shaped cells; arrow head) are positive for Ki67.

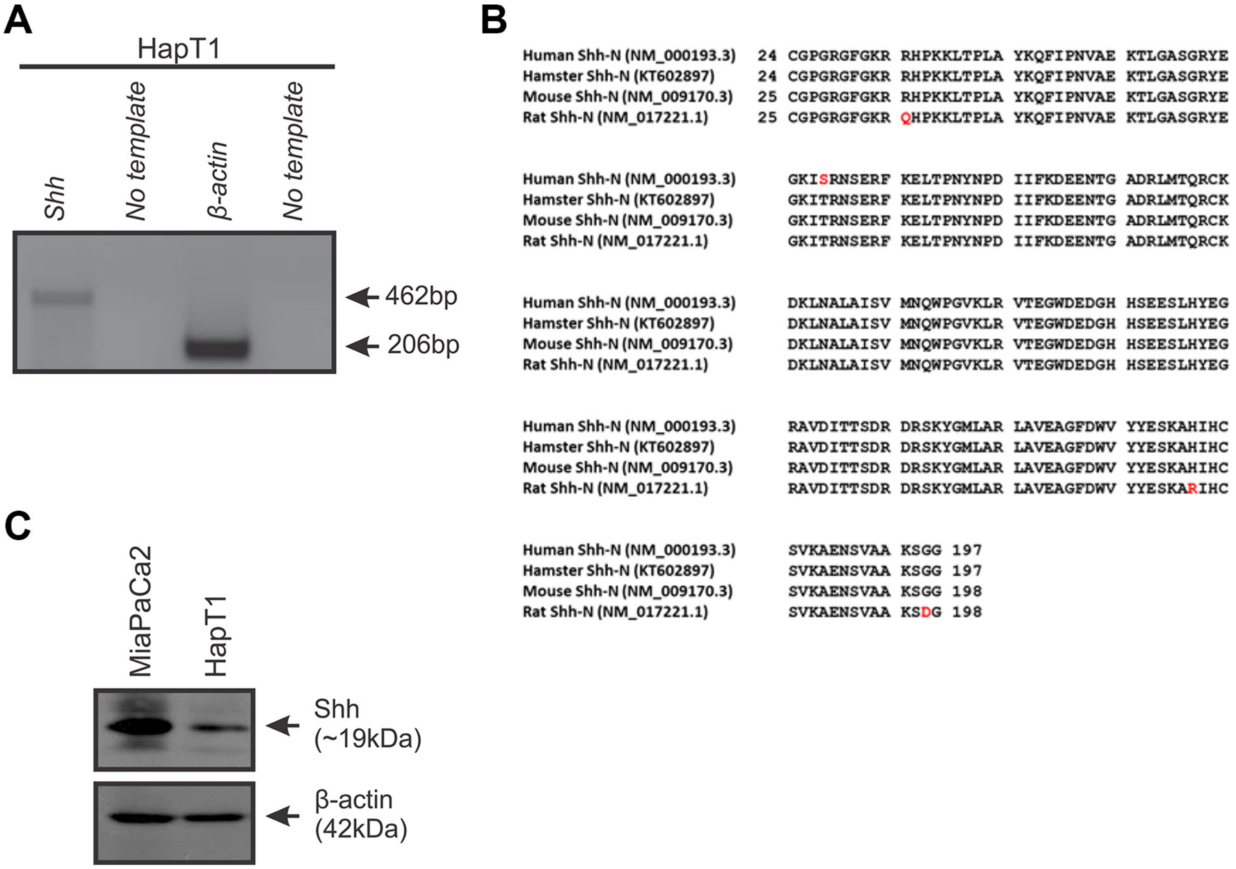

Supplementary Figure S5: Expression of Shh in HapT1 hamster-PCCs, and sequence analysis of biologically active hamster Shh. (**A**) Sonic hedgehog expression in HapT1 cells was checked through PCR analysis. Expression primers were designed from the predicted sequences available in the NCBI database (XM_005084199.2). Our PCR analysis clearly shows amplicons of expected band size (462 bp). (**B**) The amplified product covers the nucleotide sequences that encode the N-terminal Shh (the physiologically active form of Shh). We cloned this PCR product and sequenced it. The sequence analysis confirmed it as Shh and the actual nucleotide sequence completely matched with the predicted m-RNA sequence (XM_005084199.2). The sequence information was submitted to GenBank and the accession number is KT602897. Moreover, sequence alignment of the hamster N-terminal Shh amino acid sequences (aa24-194) with N-terminal Shh of other commonly used experimental animals (mouse and rat) and human reconfirmed the highly conserved nature of this region. Hamster N-terminal Shh has a 99% and 100% identity with human and mouse N-terminal Shh, respectively (non-identical residues are highlighted in red). (**C**) Importantly, reactivity of Shh antibody generated against a human N-terminal Shh peptide with hamster N-terminal Shh further indicates the similarities between these proteins.

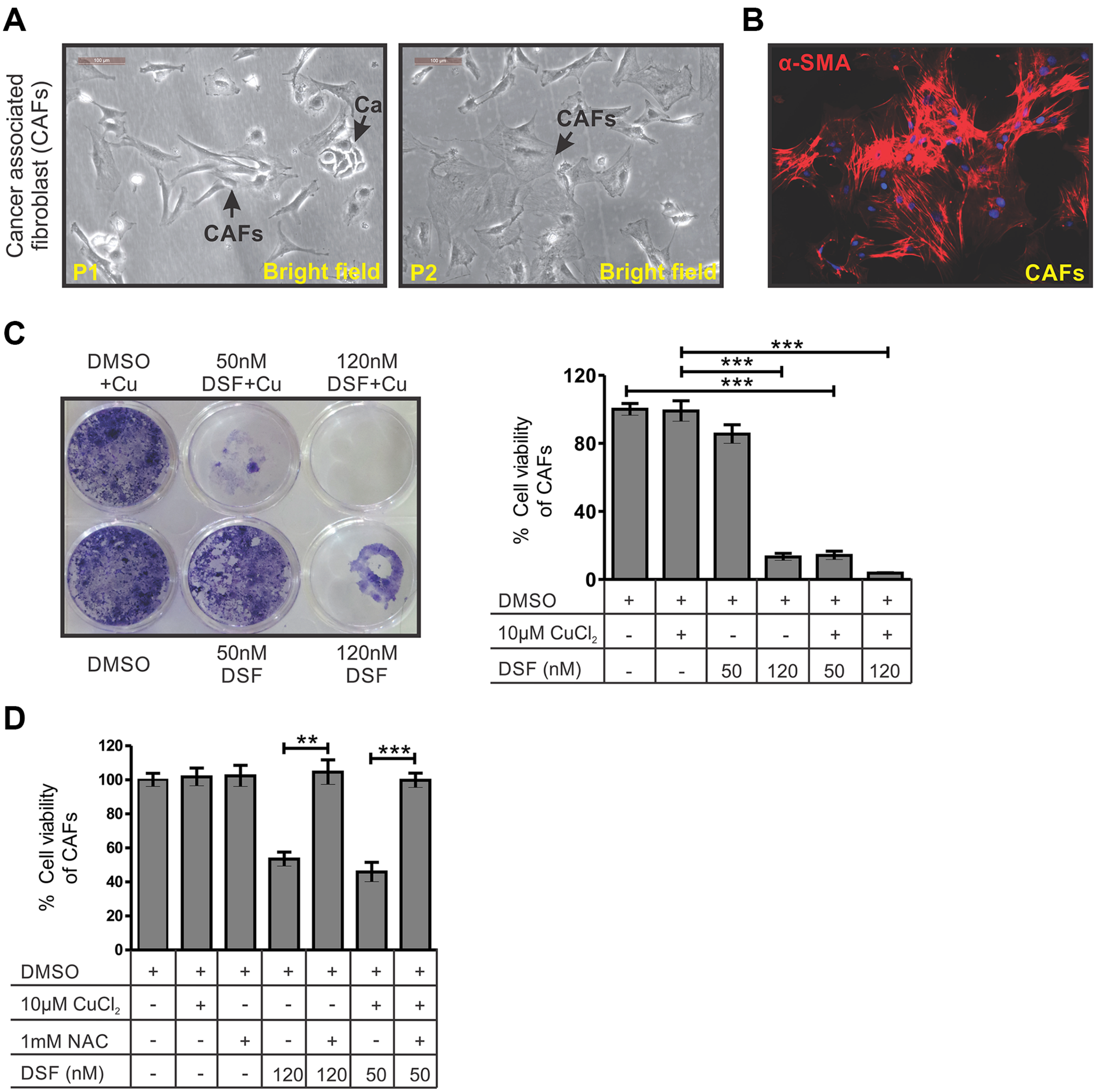

Supplementary Figure S6: Isolation, culture and characterization of HapT-1 cancer-associated fibroblast cells (CAFs) and the effect of DSF on these cells. (**A**) Bright field image of cells isolated from HapT1 primary tumors shows presence of both CAFs (isolated) and cancer cells (colonies; Ca). After 2nd passage (differential trypsinization) there was no visible cancer cell colonies in the culture plate. (**B**) Immunofluorescence staining of 3rd passage cells for α-SMA shows homogenous population of activated CAFs. (**C**) Crystal violet staining followed by quantification clearly shows the significant cytotoxic effect of 120 nM DSF and 50 nM DSF with 10 µM $CuCl^2$ on CAFs. The data presented as mean ± SE ($n = 3$) and ***$p < 0.0005$. (**D**) Cell viability checked through MTT assay shows rescue of CAFs form DSF-mediated cell death after pre-incubation with 1mM NAC. The data presented as mean ± SE; **$p < 0.005$; ***$p < 0.0005$ ($n = 3$).

Supplementary Table S1: List of primary antibodies used for immunoblot and/or immunohistochemical analysis

Sl. No.	Antibody	Company	Catalog No.	Dilution for western blotting	Dilution for IHC
01	Anti-α-SMA (Mouse Monoclonal)	Sigma	A2547	1 : 1000	1 : 2000
02	Anti-Ki67 (Rabbit Monoclonal)	Vector Laboratories	VP-RM04	NA	1 : 50
03	Vimentin (Rabbit Monoclonal)	Cell Signalling Technology	D21H3	1 : 1000	1:100
04	Fibronectin (Rabbit Polyclonal)	abcam	ab2413	1 : 1000	1 : 50
05	Shh (Goat polyclonal)	Santa Cruz Biotechnology	Sc-1194	1:1000	NA
06	Collagen-1 (Rabbit polyclonal)	abcam	Ab34710	1:1000	NA

Supplementary Table S2: List of primers

Gene Name	NCBI Reference Sequence	Primer sequence	T_m (°C)	Product length (bp)
Shh	XM_005084199.2	F: ATGCTGCTAGCGAGATGTC<br>R: GTCCCTGTCAGATGTGGTGAT	60	462
Ptch1	XM_005078436.1	F: CTCCCAAGCAAATGTATGAGC<br>R: GCATGGTTAAACAGGCATAGG	57	270
β-actin	NM_001281595.1	F: CCTCTATGCCAACACAGTGC<br>R: CCTGCTTGCTGATCCACATC	55	206
Galectin 1	XM_005066966.2	F: CAACCTGAATCTCAAACCTG<br>R: GCCACACACTTGATCTTGAA	55	375
Galectin 3	XM_005085734.2	F: ACTGGCCAGAATTTTCATGT<br>R: CACGGAGCCTTTAAATGAGT	55	865
Sparc	XM_013111769.1	F: CTTCCTGCTGCTTGTCTCTT<br>R: GCAGCTTCTGCTTCTCAGTG	55	709
Bdnf	XM_005064810.2	F: CATACTTCGGTTGCATGAAG<br>R: CCGAACATACGACTGGGTAG	55	620
Pdgfa	XM_005080002.2	F: GAGGAAGCCGAGATACCC<br>R: TCTGGGTTCAGGTTGGAG	55	500
Postn	XM_005077468.2	F: CTGCAAATGCCAACAATTAC<br>R: GCTCTGAACGATGAAAGGTC	55	703
Plau	XM_013123122.1	F: GTGGTGAACGACTCTGAAGG<br>R: GGCTCTCAATGACAGTGGAT	55	532
Annexin A2	XM_005075483.2	F: GTTTCTGGGGAGGTTTTCAG<br>R: ACCTTTCGAACACTTTCTGG	55	746
TaglnX1	XM_013112934.1	F: CCCAGGCACTGACACTACTC<br>R: TGCCCATGTACAGTCATCTT	55	816
TaglnX2	XM_013112935.1	F: ACGTCTGGGTCCTCTGAGT<br>R: CTACATAGGCGTGGGTGAG	55	736
Serpinh1	XM_005073977.2	F: GGCAGTGAGTTGAGCAGAG<br>R: GCTTAAAGAACATGGCATTG	55	725
Timp1	XM_005085712.2	F: GGCTTCTAGAGAGACACCAGA<br>R:GAAAGAATAATGGAGGGAAGG	55	716
Tagln2	XM_005078199.2	F: GGTCTTGAGCTCCACTCG<br>R: AGGGAAGCTTAGAGGTCCAG	53	800
Mif	XM_005070746.2	F: GGTCACGTAGCTCAGGTCTC<br>R: CCGTGGTCTCTAAACCATTT	55	555
Edn1	XM_005066359.2	F: CCGAGATCTCAATTAGCAGAG<br>R: GGAGAGTGCAGAGTTAGTTGG	55	811

Supplementary Table S3: Proteins identified through mass spectrometry analysis
